# The role of social–emotional competencies in interpersonal relationships: a structural equation modeling approach

**DOI:** 10.3389/fpsyg.2024.1360467

**Published:** 2024-10-03

**Authors:** Jing Lin, Letong Zhang, Yi-Lung Kuo

**Affiliations:** ^1^Collaborative Innovation Center of Assessment for Basic Education Quality, Beijing Normal University, Beijing, China; ^2^School of Information Resource Management, Renmin University of China, Beijing, China; ^3^College of Education Sciences, The Hong Kong University of Science and Technology, Guangzhou, China

**Keywords:** social–emotional competencies, structural equation modeling, interpersonal relationships, gender differences, adolescent

## Abstract

Social–emotional competencies, a crucial non-academic factor for K-12 students to be competent 21st century citizens, are receiving increasing research attention. Based on the 2,801 self-reports of grades 4–8 students from four schools in China, this study scrutinized the associations between adolescents’ five core competencies and their interpersonal relationships. Results indicated that five competencies acted as mediators between parent–child relationships and students’ relationships with teachers and peers. In addition to the girls’ advantage in five competencies, the results also demonstrated the gender and grade differences in the association. Boys had more indirect links and girls had both direct and indirect associations. There were grade differences in the mediating role of social awareness. The study’s findings may advance our understanding of the parental influence on Chinese students’ social–emotional competencies and provide a more nuanced picture of the conditions and interplay that foster or hinder students’ proficiency in these competencies in schools and families.

## Highlights

- This study examined the associations between five social-emotional competencies and three types of interpersonal relationships via analyzing cross-sectional data from Chinese students in Grades 4 through 8.
- Parent-child relationships were directly and indirectly related to teacher-student relationships for girls and boys via social-emotional competencies, except the only indirect associations mediated by self-awareness, social awareness, and relationship skills were found among boys.
- Parent-child and peer relationships were indirectly linked via the mediator of social-emotional competencies, except the only direct association existed when mediated by girls' social awareness.
- Among girls, parent-child relationships were more strongly associated with five social-emotional competencies, their teacher-student relationships, and peer relationships.
- There were grade differences in adolescents' social awareness in mediating their interpersonal relationships.

## 1 Introduction

The era has witnessed great emotional stimulus and mis/dis-information, putting individuals at increasing risk of negative mood and poor social interactions (OECD, 2021). To survive and thrive in this complex and connected world, non-academic skills such as collaboration and social skills have been embraced as important 21st century learning outcomes (Trilling and Fadel, 2009). Fostering an individual’s proficiency in core social–emotional competencies (SEC) is recognized as a promising way of framing these outcomes, thereby contributing to an individual’s academic, career, and life outcomes (Osher et al., 2016). SEC is a multidimensional construct embodied consistently in thoughts, feelings, and behaviors (OECD, 2015; CASEL, 2020). Many countries and international organizations regard SEC as a core element of human development (e.g., Guerra et al., 2014; OECD, 2015), especially for positive youth development (PYD) (Taylor et al., 2017), aiming to develop students into confident and active citizens with social responsibility and ethics (Ee et al., 2014).

The core components of SEC have also been continually emphasized in Chinese policy documents (e.g., MOE, 2021). The newly promulgated curriculum standards explicitly incorporate intellectual curiosity, cooperation, and cross-cultural understanding into the developmental goals for students (MOE, 2022). Under the circumstance of the fierce academic competitiveness in China, students’ non-academic competencies have been traditionally neglected over academics in school practices. As a result, there is an urgent need to examine the SEC proficiency of Chinese students to provide evidence-based directions for promoting practices. A recent survey conducted in Guangdong province indicated that supportive parenting practices were needed to promote preschoolers’ social and behavioral development (Wang et al., 2021). Another study conducted in western China suggested that enhancing students’ SEC might promote teacher-student relationships and academic performance (Wang et al., 2019). These two studies call for the need for more research on students’ SEC, with particular emphasis on considerations on their social interactions and environments. Given the tension between the emphasis on SEC and examination-oriented practices in China, this issue is highly critical for PYD and fostering competent 21st-century citizens.

Students’ SEC proficiency has been found to be related to many factors, including age, gender, and socioeconomic background (OECD, 2021), and the specific competencies may vary across these factors and present different developmental trends (e.g., Rodríguez-Álvarez et al., 2021). As a crucial development period, adolescence represents an important period for research (e.g., West et al., 2020; Ross et al., 2019). The present study contributes to cross-sectional comparisons of SEC across early adolescence by dealing with data spanning five consecutive grades. An important aspect of SEC is its responsivity to interpersonal interactions (Osher et al., 2016) and association with different social relationships including parent–child (Allen et al., 2018), teacher-student (Brion-Meisels and Jones, 2012), and peer relationships (Trentacosta and Fine, 2010). Whether SEC improves with age, or which gender performs better during adolescence, is not a consensus of existing research (e.g., Portela-Pino et al., 2021; OECD, 2021). Some studies (e.g., Eccles et al., 1997; Connell and Wellborn, 1991) have shown that girls benefit more than boys (e.g., achieving better academic performance) from feeling connected and supported emotionally in both parent–child and school contexts. It is still unclear whether girls also benefit more than boys from the role of SEC in interpersonal relationships. However, little research has investigated the intricate associations among the three types of interpersonal relationships and SEC in a comprehensive and structured manner. To fill the gap, this study explored the following research questions with a sample of early adolescent Chinese students:

1. What are the associations among students’ SEC and their relationships with parents, teachers, and peers? Are there mediating effects between these variables?
2. Are there grade or gender differences in the associations among students’ SEC and their relationships with parents, teachers, and peers?

## 2 Theoretical perspective

### 2.1 Definition of SEC

SEC has been referred to with different labels (e.g., non-cognitive factors, psychosocial factors, social and emotional skills) and overlaps with other relevant constructs, such as emotional intelligence (Salovey and Mayer, 1990), social and behavioral skills (DiPrete and Jennings, 2012), and interpersonal sensitivity (Murphy and Hall, 2011). SEC and emotional intelligence are not clearly distinguished in use (Lozano-Peña et al., 2021). Emotional intelligence is defined as the ability to understand and distinguish feelings and emotions and use emotional information to guide actions (Salovey and Mayer, 1990). To date, there are two major representative SEC frameworks in the fields. The Collaborative for Academic, Social, and Emotional Learning (CASEL, 2020) integrates the connotation of emotional intelligence into the term “socio and emotional competence” to describe whether and to what extent individuals are able to apply skills, attitudes, and behaviors to deal with everyday tasks and challenges in an effective and ethical manner (Elias et al., 1997). Through further categorization, the constructed competence is conceptualized as a set of abilities to interact with others, monitor and manage cognitive processes, and regulate emotions and behaviors (Rimm-Kaufman and Hulleman, 2015, for further discussion). OECD (2015) uses the term “social and emotional skills” to refer to a range of skills that enable individuals to manage goal-oriented and task-directed behaviors and regulate emotions in social situations. Despite differences in definitions, it is generally accepted that SEC involves a variety of capabilities, including understanding and accepting themselves, negotiating everyday situations, and adapting to different situations (Schoon, 2021), which are also critical developmental assets valued in the PYD frameworks (e.g., Benson and Scales, 2009). This study adopts the term “social–emotional competency” which is more commonly shown in the existing literature, and defines it as a multidimensional construct that encompasses a range of specific competencies to understand, manage, and express social and emotional aspects of people for success in various contexts.

### 2.2 Measurement models for SEC

Different approaches for measuring SEC have emerged in the past two decades. Some studies drew on emotional intelligence theory to develop measurement models (e.g., Mayer et al., 2000; Bar-On, 2006). The multidimensional constructs of SEC proposed by CASEL and OECD are widely recognized and can be applied in diverse cultural contexts to capture the crucial competencies of intrapersonal and interpersonal domains. CASEL proposed five core competencies to characterize the SEC construct, including self-awareness, social awareness, self-management, relationship skills, and responsible decision-making (CASEL, 2020). Self-awareness refers to comprehending a student’s own feelings, ideas, and values and how they impact behavior. Social awareness is about understanding others’ viewpoints and showing empathy. Self-management refers to students can successfully control their emotions, thoughts, and behaviors and fulfill their objectives. Responsible decision-making describes that students can make good personal and socially relevant choices. Relationship skills are to build and maintain positive relationships and negotiate with others. These five competencies are treated as the key objectives to point to a common vision of cultivating students to be responsible and healthy, and of preventing students from being influenced by risks such as violence or dropping out (CASEL, 2003, 2020).

OECD (2019) used the term sub-domains to address the SEC construct referring to the sub-dimensions of the Big Five model. Through specific selection criteria (e.g., being able to predict life outcomes), 15 skills have been screened from a number of candidate skills and grouped into the five sub-domains correspondingly (see Chernyshenko et al., 2018, for the detailed discussion). Ultimately, the five sub-domains and the corresponding skills are: task performance contains the skills of self-control, responsibility, and persistence; emotional regulation contains the skills of stress resistance, emotional control, and optimism; collaboration contains the skills of empathy, co-operation, and trust; open-mindedness contains the skills of curiosity, creativity, and tolerance; and engaging with others contains the skills of energy, assertiveness, and sociability. OECD evaluates students’ performance in the above 15 skills through triangulation, i.e., students’ self-reports, peer evaluations, and reports from parents and teachers (OECD, 2019).

Even with their varied constructs, the competencies of SEC the OECD and CASEL recommend are valued in the PYD frameworks. For example, the CASEL’s competencies intersect with the PYD internal assets (Benson et al., 2007), particularly in social competencies (e.g., planning and decision making, interpersonal competence); the OECD’s 15 skills overlap significantly with Benson’s five social competencies and six positive values (e.g., responsibility, restraint). These competencies of SEC are also encompassed within Lerner’s 5/6cs models of PYD (Lerner et al., 2015), where, for example, cognitive competence denotes cognitive abilities such as decision-making, and social competence points to interpersonal abilities such as the ability to conflict resolution. Furthermore, these competencies, in turn, highly overlap with Catalano’s 15 PYD constructs (Catalano et al., 2004) such as social competence, emotional competence, and cognitive competence. Thus, adolescents’ proficiency in SEC is essential for PYD and demands great attention from researchers and practitioners in basic education.

With its origins in the Big Five model, the OECD seems to prefer to view SEC as a malleable trait and its SEC construct has been criticized for embracing personality factors related to biological features and neglecting some critical skills (Jones et al., 2016). In addition, the OECD (2023) is more interested in the impact of SEC on students’ transition from school to work, productivity and job satisfaction, physical and mental health, and overall well-being. In comparison, CASEL focuses more on an educational perspective emphasizing SEC as educationally developable. CASEL (2023) accentuates that K-12 schools should take action to promote students’ SEC proficiency in school culture and academic curricula and collaborate with communities and families, with the goal of educational equity and excellence. Since our primary interest is to improve school practices rather than the workforce, the multidimensional construct of SEC advocated by CASEL is adopted in this study.

### 2.3 Demographic factors related to SEC

SEC can change across the lifespan (Malik and Marwaha, 2022). Socialization and learning shape individuals’ SEC, and contextual factors lead to the specific development of SEC. Numerous studies have shown that age, gender, and family SES are associated with the development of students’ SEC (e.g., Liu Q. et al., 2020; Nakajima et al., 2020; West et al., 2020).

#### 2.3.1 Age

SEC changes as individuals mature biologically (Feraco and Meneghetti, 2023). For example, the prefrontal cortex continuously develops until age 25, which aids decision-making and controls impulses and emotions (Arain et al., 2013). Individuals of all ages have different social and emotional needs that trigger different behaviors, leading to different aspects of SEC proficiency (Osher et al., 2016). For example, pre-adolescents develop basic empathy and perspective taking, as they capture and understand subtle surrounding information to support better social interactions (Ross and Tolan, 2018); for adolescents, many students turn to more specific interpersonal skills, such as resolving conflict and engaging in prosocial and ethical behaviors (West et al., 2020). Among adolescents aged 10–18, their self-awareness showed a decreasing and then increasing trend of change, reaching its lowest around age 16, while relationship skills exhibited an increasing and then decreasing trend of change, reaching its highest around age 15, with both showing a quadratic trend of change (Ross et al., 2019). Even when looking at SEC as a whole, there is no consensus on how SEC changes in adolescence, although it is generally accepted that SEC varies with age. A survey investigating students aged 11 to 18 confirmed that older students presented better SEC (Portela-Pino et al., 2021). On the contrary, according to OECD (2021), older students were more likely to perform worse than younger students on many skills of SEC, including tolerance and empathy.

#### 2.3.2 Gender

Gender has been a factor recognized for an association with certain competencies but not for the overall construct of SEC (Ross et al., 2019). Girls may outperform boys to varying degrees in many abilities, such as responsible decision-making, social awareness, and relationship skills (DiPrete and Jennings, 2012; Nakajima et al., 2020), while girls report lower self-efficacy and emotional regulation skills (West et al., 2020). For certain competencies, gender differences are less clear. Rodríguez-Álvarez et al. (2021) demonstrated girls’ higher level of self-management than boys’, but in another survey, boys performed better in self-management (Portela-Pino et al., 2021). The development trajectories of each competency might differ for boys and girls, and the gender gap could narrow overall at the end of adolescence (Ross et al., 2019). However, compared to other cities, Suzhou (China) and Daegu (Korea) had slight gender differences for younger students but much more pronounced differences for older students (OECD, 2021). This is likely due to Asian cultural norms and different socialization, in which males are more expected to expand their social network, while females are expected to provide more family care (e.g., Zhu et al., 2020). In short, the gender differences in SEC, which also involve differences in individual maturity, are complex and not yet conclusive.

#### 2.3.3 Family SES

A large body of research has supported the links between family SES and students’ SEC (e.g., Kuo et al., 2020). In the Survey of Social and Emotional Skills, student’s family SES was positively related to SEC (OECD, 2021). Adolescents from disadvantaged families may have lower levels of SEC (Gershoff et al., 2007). Though low family SES might lead to worse SEC such as lower emotional resilience, positive parent–child relationships may alleviate the negative impact of low family SES (Cao et al., 2021). Family SES might also be linked to perceived peer relationships by affecting students’ self-esteem and stress (Bai et al., 2021). Students who lack financial resources might have fewer opportunities to participate in social activities, less confidence in interpersonal interactions, and worse peer acceptance (Matthews et al., 2015).

### 2.4 Interpersonal relationships associated with students’ SEC

Children’s SEC develops in interactions with formal and informal settings and is highly responsive to interpersonal interactions (Osher et al., 2016). Pre-school children primarily interact with family members and gain socialization experiences. Once in school, teachers and peers become important social groups and can increasingly play a vital role in shaping students’ SEC (Allen et al., 2018). In the process of these interactions, interpersonal relationships are the key drivers for student SEC development (Osher et al., 2018; Jones and Bouffard, 2012). Meanwhile, students’ SEC proficiency is related to the quality of these relationships, including parent–child, teacher-student, and peer relationships (Wang et al., 2019). There may be a bidirectional relationship between interpersonal relationships and SEC.

#### 2.4.1 Parent–child relationships

The influence of parent–child relationships on SEC forms by parenting practice (Kelly et al., 2024; Landry et al., 2006). Supportive parenting practices were positively linked to children’s social functioning (Boeldt et al., 2012). For example, a study in Guangdong province showed that supportive maternal parenting, including warmth and responsiveness, emotional availability, and encouragement, was positively related to children’s social skills and negatively to their problem behaviors (Wang et al., 2021). By surveying a sample of 965 adolescents from Hong Kong, China, Zhu (2019) found the associations between parenting behaviors and adolescents’ SEC as well. The low SEC in early childhood may stem from the poor quality of parent–child relationships, and the distrustful, unreliable, and unsupportive relationships can negatively impact students’ psychological, social, and emotional development (Tehrani et al., 2024; Thompson, 2005). Students who lack secure connections with parents may experience great difficulty in developing SEC (Allen et al., 2018). Meanwhile, Chinese students had less decision-making autonomy and greater reliance on parental authority than American students (Qin et al., 2009). Thus, parent–child relationships may play a solid role in the associations with SEC and even other interpersonal relationships among Chinese adolescents.

#### 2.4.2 Teacher-student relationships

Teacher-student relationships are positively correlated with students’ SEC (Wang et al., 2021). For one thing, students in good relationships with teachers generally had access to meaningful interactions (Brion-Meisels and Jones, 2012), receiving the critical support they needed (Rucinski et al., 2018). When students received more respect and care from their teachers, they were inclined to have higher levels of self-esteem and self-identity (Jose et al., 2012). Students with high SEC tended to seek help from teachers when they encountered difficulties in learning, so their relationships with the teacher were more positive (Zins et al., 2004). Many studies have shown that the SEC was a positive predictive factor in teacher-student relationships (e.g., Brown and Conroy, 2011). A survey in western China found that SEC was positively related to the relationships between fourth-and fifth-grade students and teachers in various disciplines, including reading, math, and science (Wang et al., 2019).

#### 2.4.3 Peer relationships

In adolescence, peers play an increasingly vital role in students’ SEC development (Allen and Loeb, 2015). Adolescents tended to seek acceptance and connection from their peers, thereby increasing the probability of peers’ influence (Hawley et al., 2002). Students with high SEC could make good friends and demonstrate appropriate behaviors when interacting with others (Zins and Elias, 2007; Allen et al., 2018). Studies have confirmed that high SEC was positively associated with good peer relationships (Trentacosta and Fine, 2010), because students with higher SEC were usually better at communicating, listening to others, and negotiating conflict constructively (McCabe and Altamura, 2011). Students with high SEC also tended to seek and offer help when needed, which helped foster relationships among students with different academic achievements (DeLay et al., 2016).

### 2.5 The mediating role of SEC

Although a growing body of research has investigated the associations between students’ interpersonal relationships and their SEC, less empirical attention has been directed to the mediating processes underlying the associations. A child with a secure attachment history in the family was more likely to be socially competent (Cassidy and Shaver, 1999) and approach teachers with positive expectations and attitudes, possibly resulting in positive teacher-student relationships. Zhang (2011) found that preschoolers’ social competency fully mediated the association between parent–child and teacher-student relationships. For adolescents, however, it is unclear whether SEC plays the same role (Yang et al., 2024). Existing literature shows when parents interact with children in a warm and supportive manner, children may develop the competency to effectively resolve conflicts; when parents are hostile and aggressive, children may learn negative interaction patterns and build conflicting relationships with others (Myers and Pianta, 2008). Similarly, Cook et al. (2012) demonstrated that parental hostility and psychological control were significantly related to adolescents’ friendship skill, which affected adolescents’ interaction with peers and the establishment of healthy peer relationships. Hence, a conjecture can be made that children’s social cognitive skills acquired in interactions with their parents would be carried over to other social interactions, suggesting the potential mediating role of SEC in the association among different social relationships (Parke and Buriel, 1998; Perry et al., 2018). Additionally, a previous study showed that the association between parent–child relationships and SEC might differ by gender (Zhu, 2019). However, there is a lack of evidence which indicates gender or age plays a role in the relationship between interpersonal relationships and SEC.

### 2.6 The present study

**Figure 1:**
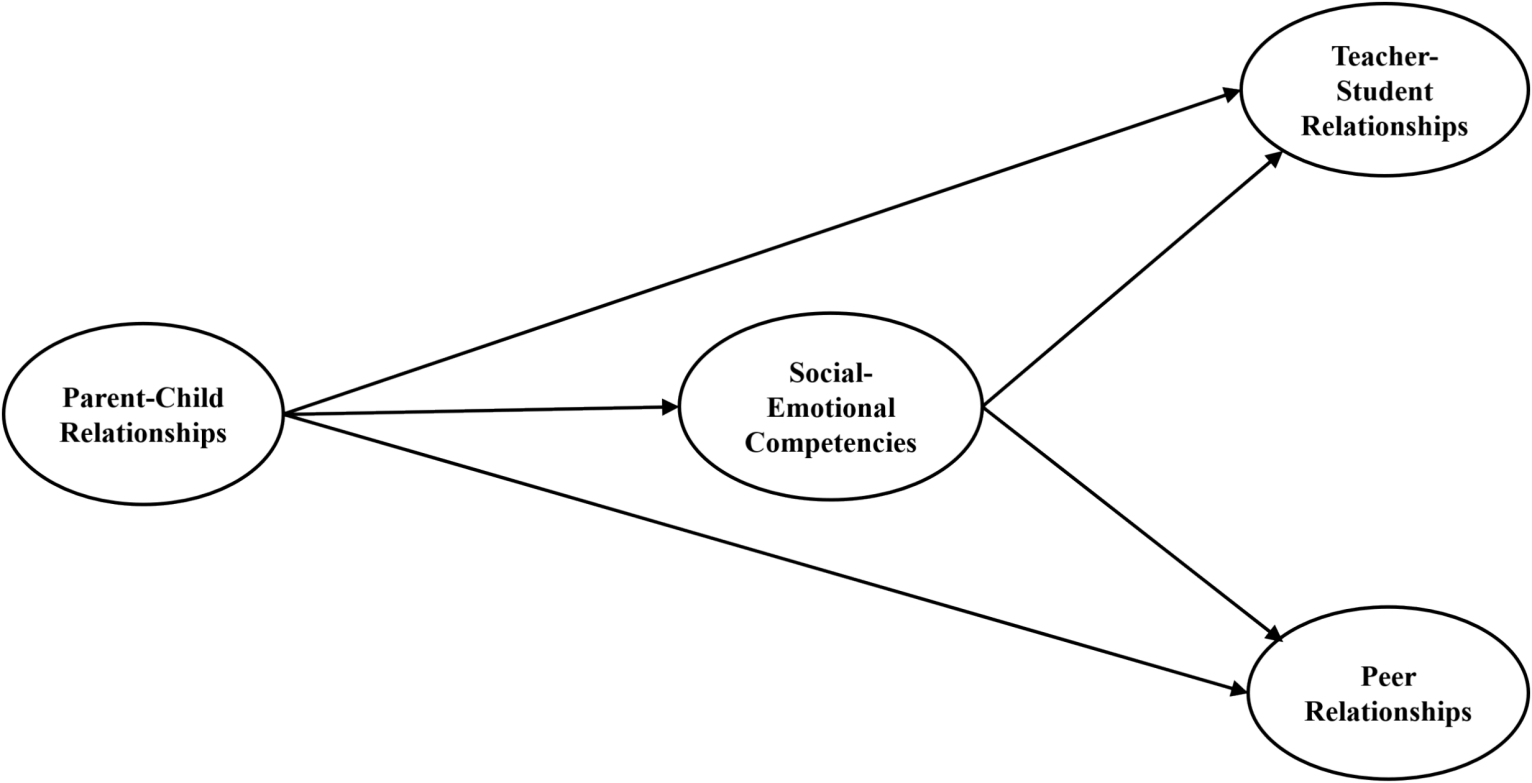
Hypothetical structural model for SEC and interpersonal relationships.

It has been suggested that children’s competencies might mediate the associations between parent–child and teacher-child relationships (Parke and Buriel, 1998). The findings of Zhang’s study in 2011 have confirmed the mediator role of social competency in interpersonal relationships among preschoolers. And in Chinese culture, parents tend to have a very strong influence on their children’s social relationships (Ng et al., 2014). The association between parent–child relationships and teacher-student relationships has been demonstrated among Chinese students in grades 4–5 (Yang et al., 2024), and the association between parent–child relationships and peer relationships has been confirmed among Chinese students in grades 4–9 (Liu L. et al., 2020). Drawing on the existing research, in this study, we would like to further correlate the associations between the three types of interpersonal relationships that students perceived and their five competencies proposed by CASEL through Chinese primary and middle school students. We assumed that adolescents’ competencies may play mediator roles in associating parent–child relationships with teacher-student and peer relationships. Figure 1 shows the hypothetical model used in this study. We conjectured that children’s competencies initially developed primarily in interactions with parents in the parent–child relationships and then migrated to the processing of relationships with others. As children develop competencies over time, the links to interpersonal relationships may be highly complex and multidirectional. The present study first tested the hypothesis that parent–child relationships are related to teacher-student and peer relationships through the mediation of SEC. Family SES might also be associated with different interpersonal relationships (e.g., Matthews et al., 2015), so SES was included in the model as a controlled variable. Further, we examined the potential effects of gender or age on the mediating role of SEC among the associations between parent–child relationships and students’ relationships with teachers and peers.

## 3 Methods

### 3.1 Sample and procedures

Participants were recruited from an eastern coastal city in China, under the assumption that they would share some regional features and policy contexts (e.g., the 5 years of elementary school plus the 4 years of middle school compulsory education system). Four public schools voluntarily participated in this study: one primary and one middle school in both urban and suburban areas, respectively. Data were collected in June 2022. Due to the high school entrance exam preparation for the ninth graders and the possible challenges in reading and understanding texts for students in the third grade and below, students from grades 4 to 8 were invited to voluntarily participate in the survey. A total of 2,887 students consented to the research and responded to the questionnaire anonymously. All measures and procedures were approved by the Research Project Ethical Review Board at the corresponding author’s institution.

The participants accessed the online questionnaire through a link provided by their teachers. The questionnaire contained three sections: demographics information, five competencies of SEC, and three types of interpersonal relationships. All participants completed the questionnaire independently at school using devices such as cell phones or tablets. The average response time was about 15 min.

The participants who did not provide background information on SES (*N* = 21) and school (*N* = 14) were excluded from the sample. Besides, the responses with SD = 0 (which means each of these participants responded identically to every individual item) were identified as invalid answers (*N* = 51) and excluded from the analysis. After the screening, a total of 2,801 responses (age mean = 12.4, min = 9.5, and max = 15.4) were included in the analytic sample with a 97.0% valid rate. There was no missing value in the final valid sample. Approximately half of the students were male (53%) and predominantly 7th graders (25.5%), with the remaining grade breakdown being 6th graders (22.4%), 5th graders (19.2%), 4th graders (18%), and 8th graders (14.9%).

### 3.2 Measures

#### 3.2.1 Demographics

A demographic questionnaire was used to gather information about gender, date of birth, grade, and family SES. Family SES was measured by three indicators, including home possessions, parents’ occupations, and parents’ education levels. The composite score of family SES was obtained by averaging the standardized scores of the three indicators (OECD, 2020; see Supplementary material 1 for more details). For students with missing data on one of the three indicators, the missing one was imputed by regression on the other two. If there was missing data on more than one indicator, the family SES was not computed and assigned a missing value. The range of family SES in this study was from −2.67 to 3.15.

#### 3.2.2 Five competencies of SEC

SEC consisted of five competencies across diverse situations. Four competencies (i.e., responsible decision making, relationship skills, self-management, and social awareness) were assessed by the DSECS-S (Mantz et al., 2018) with three items for each competency. Another one, self-awareness was assessed by the Chinese adapted version of DSECS-S (Chen et al., 2021) with five items. Items focused on addressing students’ behavioral performance rather than conceptual thoughts and were contextualized in students’ daily lives. For example, the item “I get along well with others” was used to measure students’ relationship skills. All 17 items were four-point Likert-type ranging from 1 = “not like me at all” to 4 = “very much like me” (see Supplementary material 2 for more details of items).

These items were translated and proofread by two researchers. Interviews with 10 students (two from each grade of 4–8) were conducted to reduce the ambiguity of expression and make it more in line with the Chinese context. According to the results of the pre-test (*n* = 412), the item-total correlations were all higher than 0.4 indicating high internal consistency except that one item (i.e., “I blame others when I’m in trouble”) had a low item-total correlation (*r* = 0.22). The exceptional item was removed, and 16 items were used in the main study (*α* = 0.93). All five competencies had acceptable internal consistency: responsible decision-making (*α* = 0.74), relationship skills (*α* = 0.68), self-management (*α* = 0.77), social awareness (*α* = 0.70), and self-awareness (*α* = 0.88). To avoid the non-positive definite matrix caused by collinearity among latent variables, the scores of the five competencies were transformed into factor scores by principal component analysis. The total SEC score was a composite of these factor scores with good internal consistency and evidence of construct validity [*α* = 0.91, χ^2^(5) = 124.73, RMSEA = 0.09, CFI = 0.99, TLI = 0.97].

#### 3.2.3 Three types of interpersonal relationships

Students’ feelings about how they got along with their parents, teachers, and peers (i.e., three types of perceived interpersonal relationships) were measured. These interpersonal relationships were assessed with the student questionnaire of the 2019 OECD Survey of Social Emotional Skills (SESS). The scales of parent–child, teacher-student, and peer relationships had 3, 3, and 4 items, respectively. For example, the item “I get upset easily with my parents” served to assess the parent–child relationships (see Supplementary material 3 for more details about items). All items were four-point Likert-type (1 = almost never or never true, 2 = sometimes true, 3 = often true, and 4 = almost always or always true). Similar to the SEC scale, all items were translated into Chinese and modified based on the interviews with students. After the pre-test, all ten items were retained. In the main study, there were good internal consistencies: teacher-student relationships (*α* = 0.81), parent–child relationships (*α* = 0.82), and peer relationships (*α* = 0.82). All reverse items were re-coded. In the data analysis, the higher score indicated more positive relationships with teachers, parents, and peers.

### 3.3 Data analyses

First, a preliminary analysis was conducted to test the correlation of gender, grade, family SES, three types of interpersonal relationships, and five competencies. Second, for RQ1, structural equation modeling was conducted. Mediation models were established with parent–child relationships as an independent variable, the total SEC and its five competencies as mediating variables, and teacher-student relationships and peer relationships as dependent variables. Family SES was added to the models as a control variable. The Comparative Fit Index (CFI), the Tucker-Lewis Index (TLI), and the Root Mean Square Error of Approximation (RMSEA) were used as indices for the goodness-of-fit test. The values of CFI and TLI indicated an acceptable fit at values over 0.90 and a good fit at values over 0.95 (Hu and Bentler, 1999). RMSEA was acceptable at values below 0.06 (Hu and Bentler, 1999). The bootstrapping method with 5,000 replicates was used to test the indirect effect in the models for the better power of the test (Fritz and MacKinnon, 2007). If zero were not in the 95% confidence interval (95% C. I.), it would be concluded that the effect exists at the significance level of 0.05 (Preacher and Hayes, 2008).

For RQ2, independent sample *t*-test, one-way ANOVA and regression analysis were conducted to examine individual differences. Based on the mediation model and individual difference test, the gender and grade differences were explored by multi-group analysis. The measurement invariance of the latent constructs was verified. Configural invariance was tested by estimating the unconstrained models, and then metric invariance was tested by constraining item loadings. After the measurement invariance was established, structural paths were freely estimated in different cohorts and constrained to be equal to test the differences in the path. Due to the large sample, ΔCFI was used instead of Δχ^2^. The model changed significantly across cohorts when ΔCFI was bigger than 0.01 (Cheung and Rensvold, 2002). All analyses of structural equation modeling and multi-group analysis were conducted by SPSS 25.0 and M*plus* 8.3 (Muthén, 2017).

## 4 Results

### 4.1 Bivariate correlation analysis

As shown in Table 1, the three types of interpersonal relationships had moderate correlations with five competencies (*r* ranged from 0.15 to 0.50) and among them peer relationships had the strongest correlation with the total SEC and five competencies. Slight negative correlations existed between gender and the total SEC, self-awareness, relationship skills, and peer relationships. Slight positive correlations were found between grade and social awareness, teacher-student relationships, and peer relationships. However, a slight negative correlation was found between grade and parent–child relationships. There were correlations between family SES and all variables with small effect sizes (*r* ranged from 0.10 to 0.15).

**Table 1:** Descriptive statistics and correlations of gender, grade, family SES, total SEC and five competencies, and three types of interpersonal relationships.

	1	2	3	4	5	6	7	8	9	10	11	12
1. Gender												
2. Grade	−0.03											
3. Family SES	−0.01	0.26^***^										
4. Total SEC score	−0.06^**^	0.03	0.15^***^									
5. Self-awareness	−0.05^*^	0.03	0.14^***^	0.90^***^								
6. Social awareness	−0.12^***^	0.06^**^	0.10^***^	0.80^***^	0.69^***^							
7. Self-management	−0.04	−0.02	0.12^***^	0.89^***^	0.67^***^	0.66^***^						
8. Relationship skills	−0.05^**^	0.02	0.14^***^	0.83^***^	0.74^***^	0.64^***^	0.75^***^					
9. Responsible decision-making	−0.03	0.04	0.13^***^	0.86^***^	0.59^***^	0.65^***^	0.66^***^	0.68^***^				
10. Parent–child relationships	−0.03	−0.06^**^	0.10^***^	0.28^***^	0.25^***^	0.15^***^	0.31^***^	0.21^***^	0.24^***^			
11. Teacher-student relationships	−0.03	0.09^***^	0.13^***^	0.26^***^	0.26^***^	0.23^***^	0.21^***^	0.25^***^	0.23^***^	0.13^***^		
12. Peer relationships	−0.04^*^	0.04^*^	0.14^***^	0.48^***^	0.45^***^	0.34^***^	0.40^***^	0.50^***^	0.39^***^	0.14^***^	0.28^***^	
*M*	0.53	6.00	0.00	0.00	0.00	0.00	0.00	0.00	0.00	0.00	0.00	0.00
*SD*	0.50	1.33	0.81	1.00	1.00	1.00	1.00	1.00	1.00	1.00	1.00	1.00

^*^p < 0.05, ^**^p < 0.01, ^***^p < 0.001.

### 4.2 Individual difference in SEC

Table 2 shows the results of independent sample t-tests, one-way ANOVA, and regression analysis. Gender differences were found in the total SEC, self-awareness, social awareness, and responsible decision-making. The effects (Cohen’s *d*) were slight, with the effect sizes ranging from 0.09 to 0.23 (Cohen, 1988). With the exception of social awareness [*F*(4, 2,796) = 2.73, *p* = 0.028], one-way ANOVA results indicated no grade differences in the total SEC and the five competencies. Regression analysis indicated that family SES was positively related to the total SEC and the five competencies. The effects were slight, with the effect sizes (*R*^2^) ranging from 0.01 to 0.02 (Cohen, 1988).

**Table 2:** Difference test of total SEC and five competencies.

Competencies	*t_gender_* (2,801) (Cohen’s *d*)	*F_grade_* (4, 2,796) (η^2^)	*F_SES_* (1, 2,799) (*R*^2^)
Total SEC score	3.27^**^ (0.12)	1.72 (0.002)	64.84^***^ (0.02)
Self-awareness	2.41^*^ (0.09)	2.37 (0.003)	59.01^***^ (0.02)
Social awareness	6.14^***^ (0.23)	2.73^*^ (0.004)	29.96^***^ (0.01)
Self-management	1.91 (0.07)	2.00 (0.003)	41.94^***^ (0.02)
Relationship skills	2.72^**^ (0.10)	0.56 (0.001)	54.91^***^ (0.02)
Responsible decision-making	1.36 (0.05)	1.16 (0.002)	45.25^***^ (0.02)

^*^p < 0.05, ^**^p < 0.01, ^***^p < 0.001.

### 4.3 Mediation models

**Figure 2:**
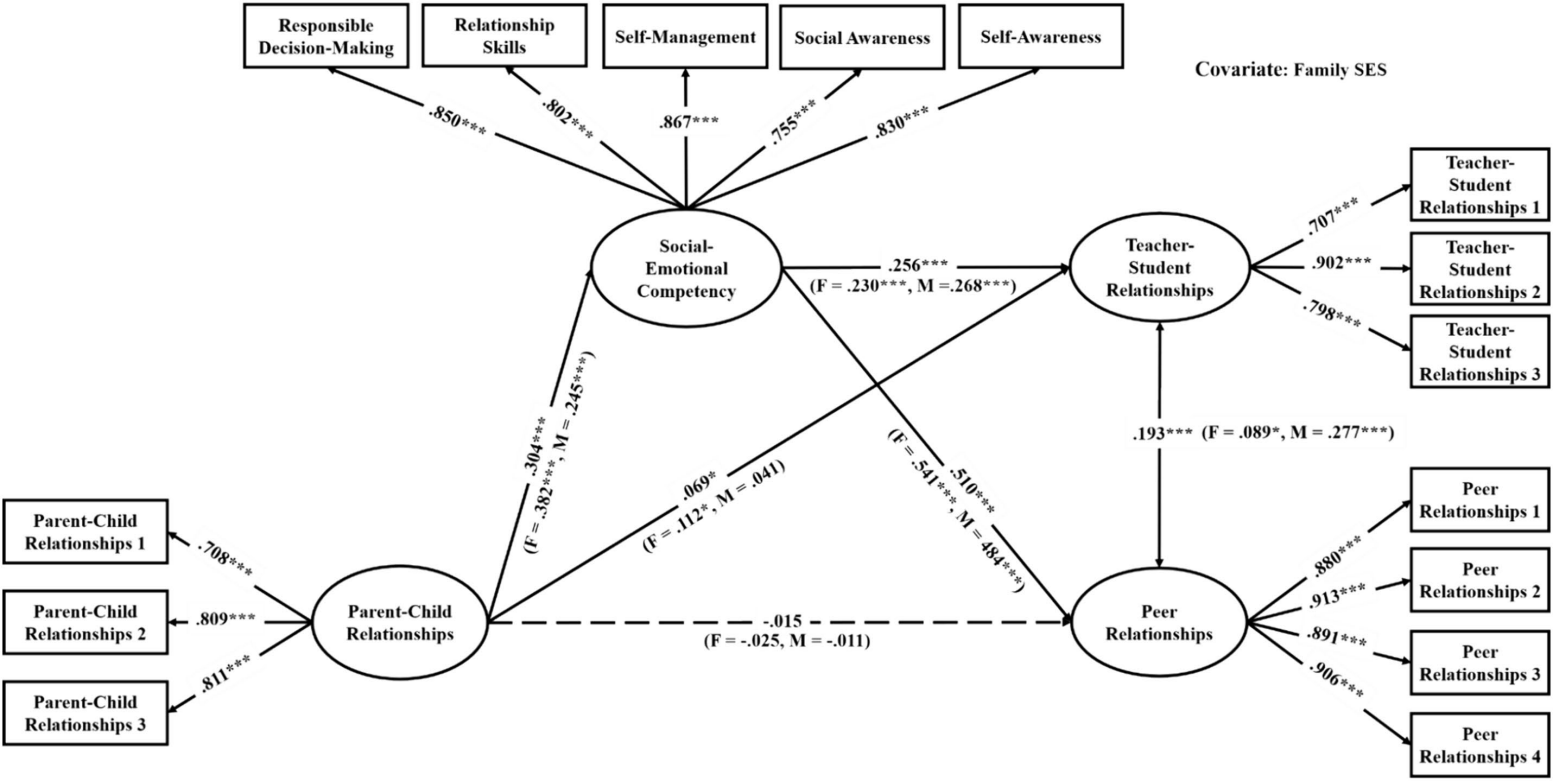
Multi-group analysis results of the indirect effect model with the mediator of total SEC (Model 1). ^*^p < 0.05, ^**^p < 0.01, ^***^p < 0.001. The standardized parameters of female (F) and male (M) cohorts are shown in the brackets next to the paths respectively.

**Figure 3:**
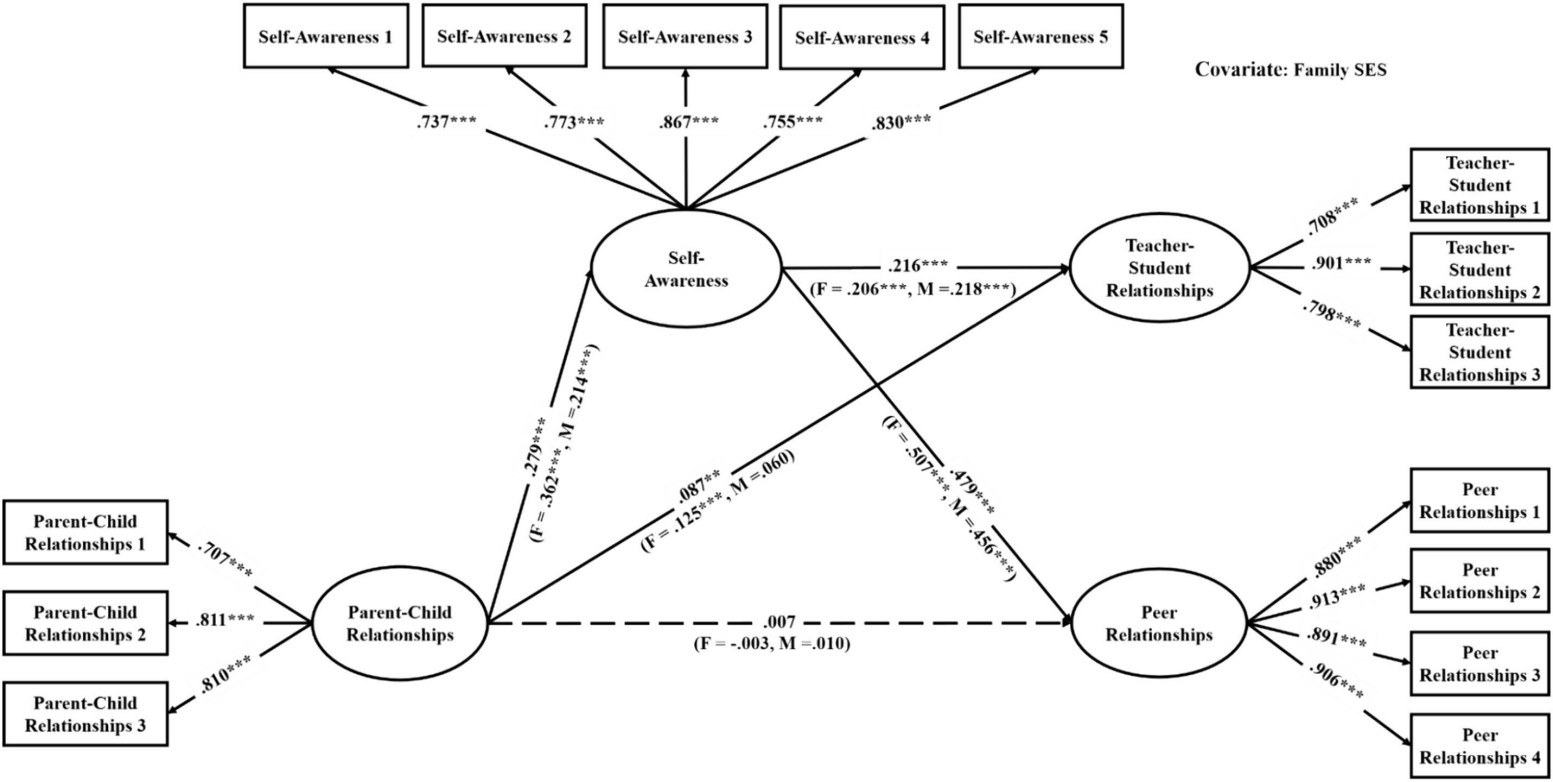
Multi-group analysis results of the indirect effect model with the mediator of self-awareness (Model 2). ^*^p < 0.05, ^**^p < 0.01, ^***^p < 0.001. The standardized parameters of female (F) and male (M) cohorts are shown in the brackets next to the paths respectively.

**Figure 4:**
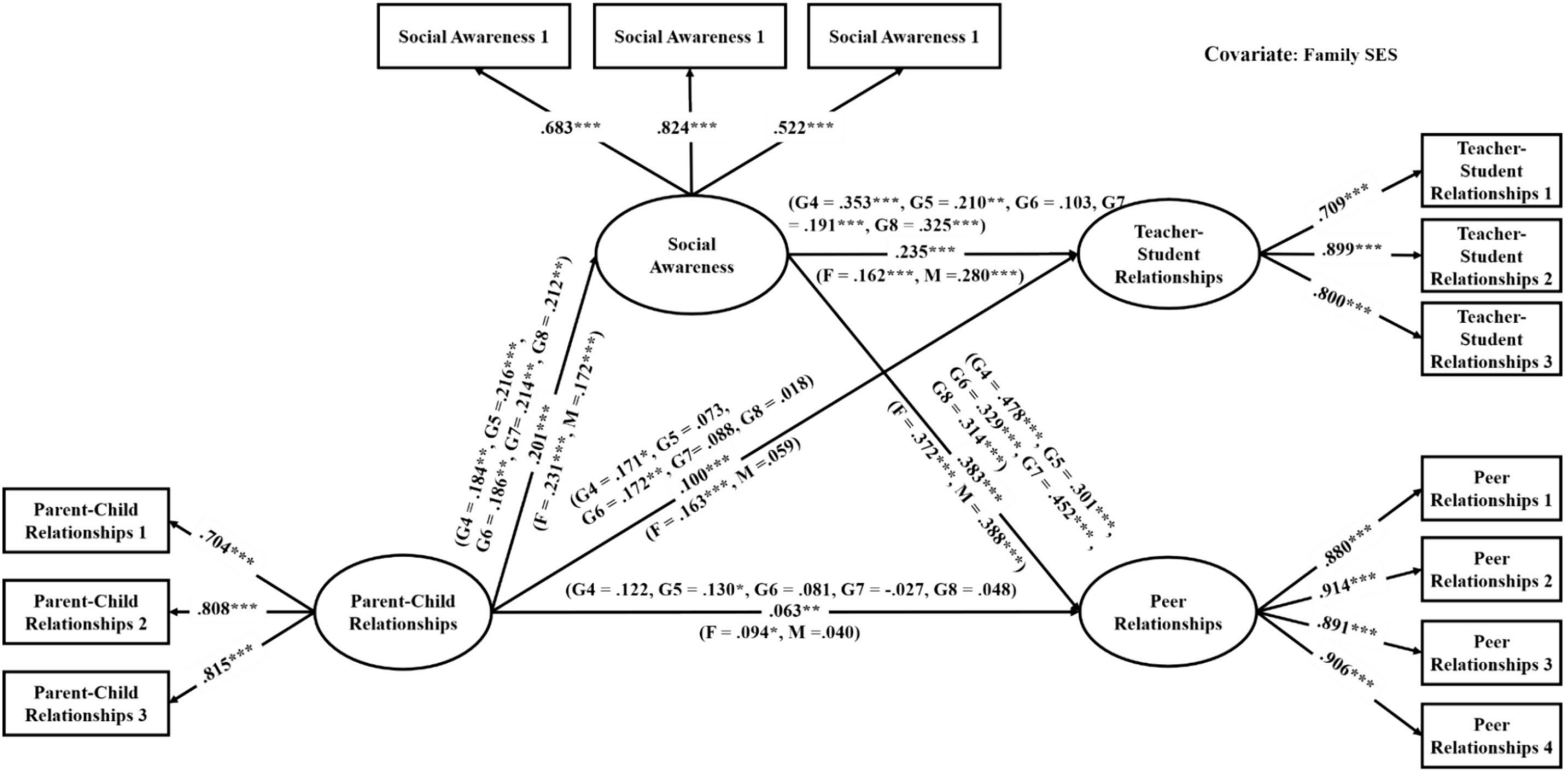
Multi-group analysis results of the indirect effect model with the mediator of social awareness (Model 3). The standardized parameters of grade 4 (G4), grade 5 (G5), grade 6 (G6), grade 7 (G7), and grade 8 (G8) cohorts are shown in the brackets above the paths, respectively. ^*^p < 0.05, ^**^p < 0.01, ^***^p < 0.001. The standardized parameters of female (F) and male (M) cohorts are shown in the brackets next to the paths respectively.

**Figure 5:**
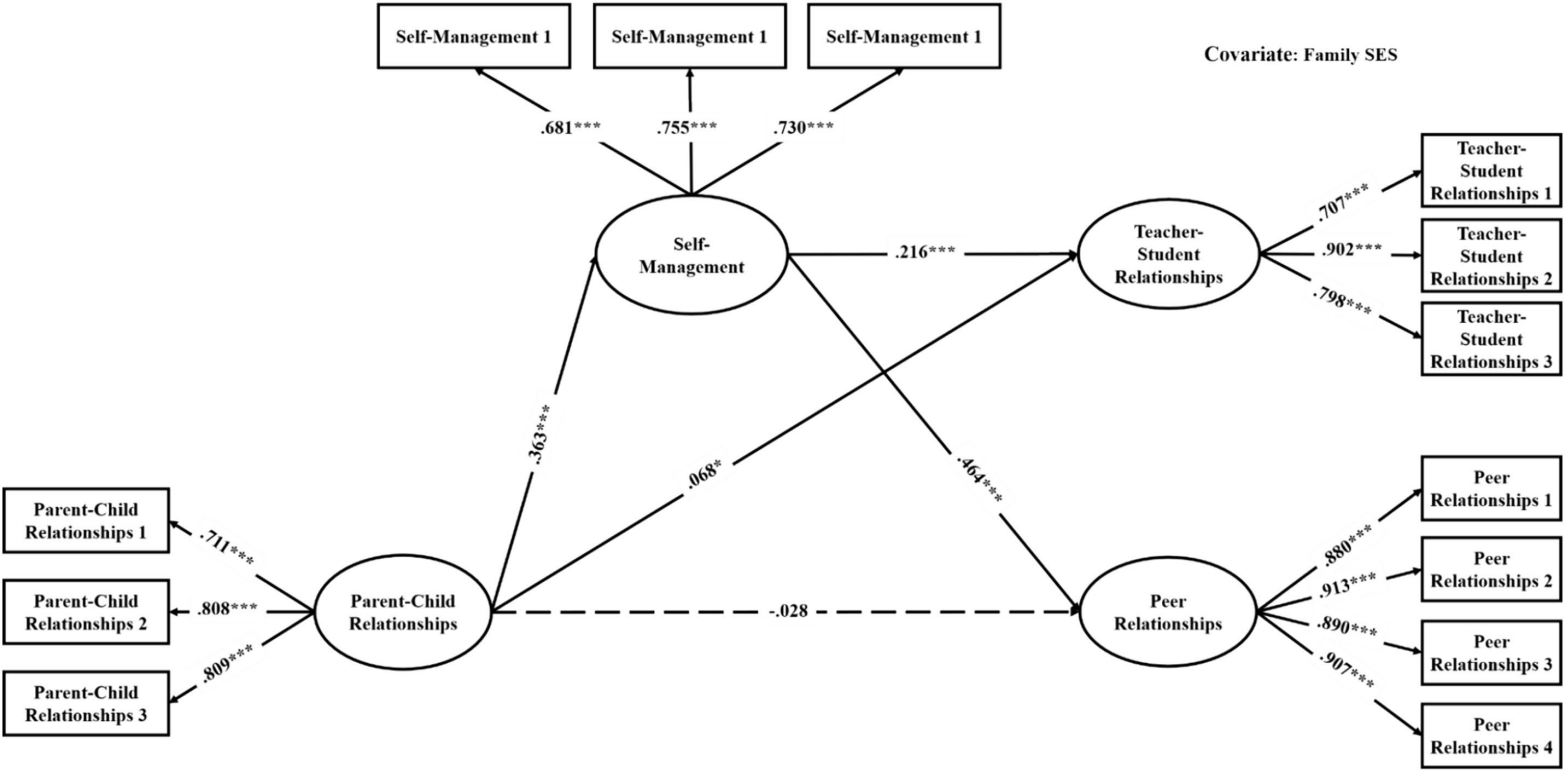
Mediation model with the mediator of self-management (Model 4). ^*^p < 0.05, ^**^p < 0.01, ^***^p < 0.001.

**Figure 6:**
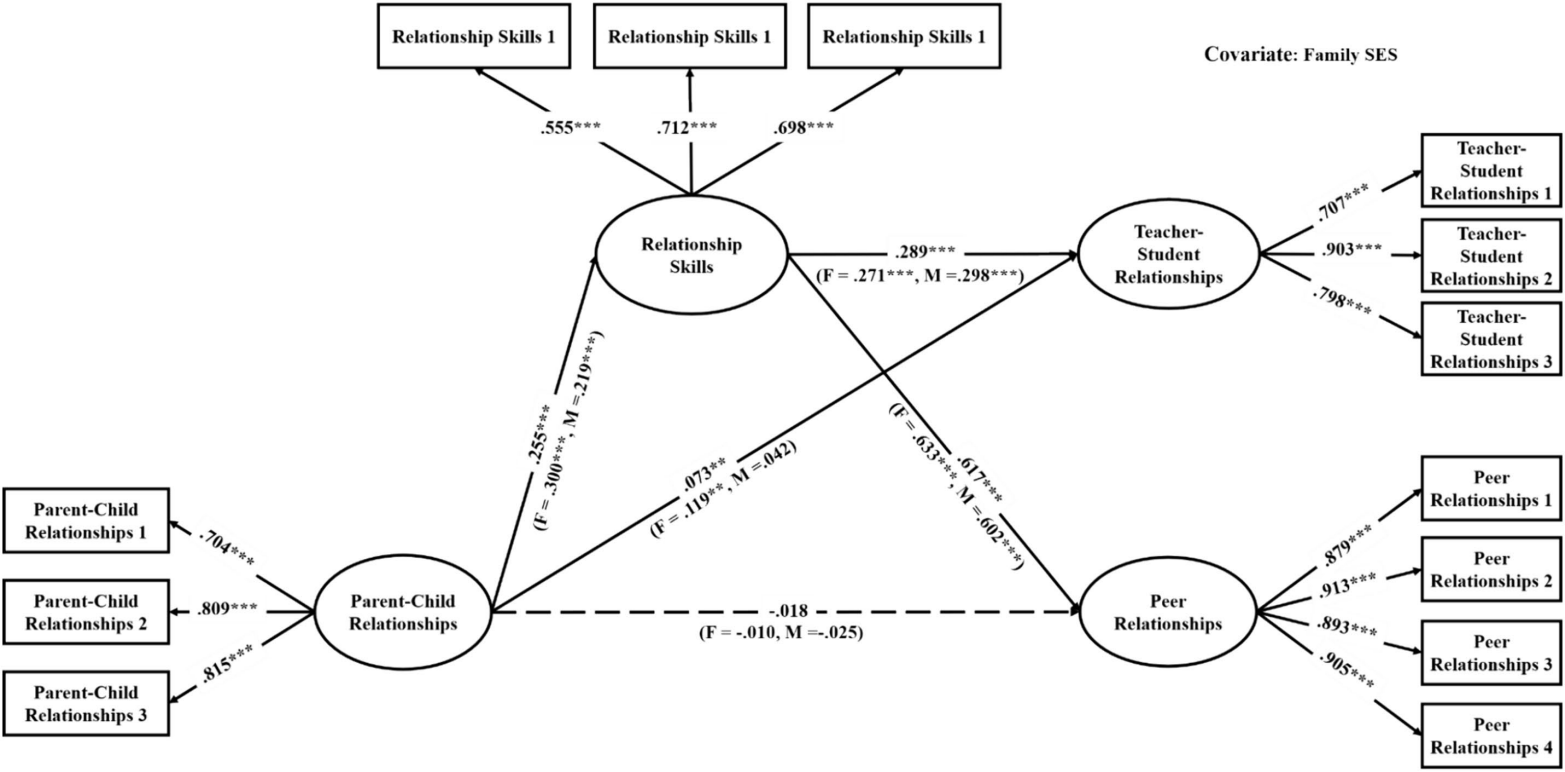
Multi-group analysis results of the indirect effect model with the mediator of relationship skills (Model 5). ^*^p < 0.05, ^**^p < 0.01, ^***^p < 0.001. The standardized parameters of female (F) and male (M) cohorts are shown in the brackets next to the paths respectively.

**Figure 7:**
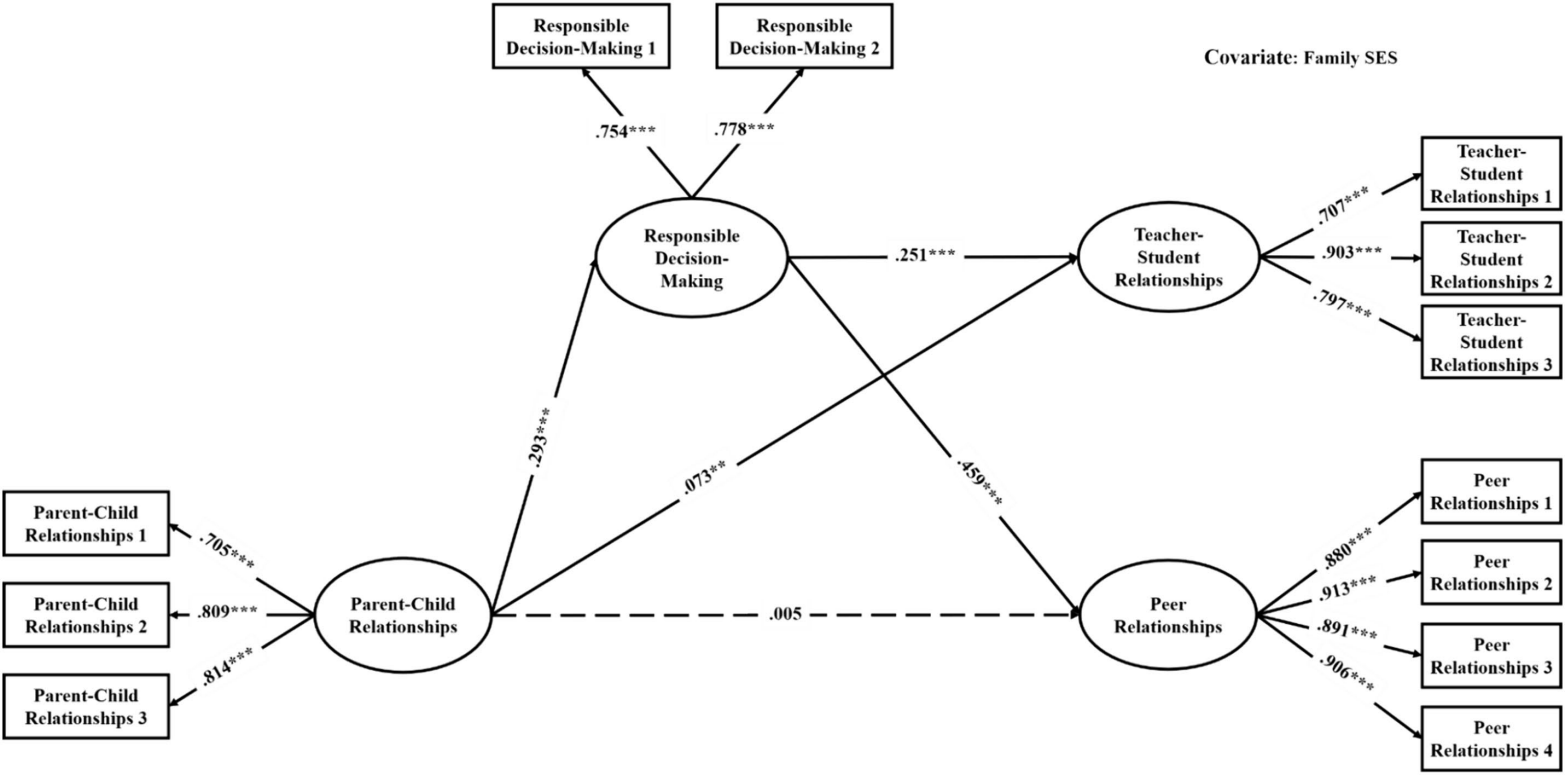
Mediation model with the mediator of responsible decision-making (Model 6). ^*^p < 0.05, ^**^p < 0.01, ^***^p < 0.001.

The mediation models were verified with good model fit (see Table 3). As shown in Table 4, among the direct links, parent–child relationships had significant associations with teacher-student relationships in all models (effects ranged from 0.06 to 0.10, see Figures 2–7). With the exception of Model 3 with social awareness as the mediator (see Figure 4), there was no direct association between parent–child relationships and peer relationships in the models. The indirect associations between parent–child relationships and peer relationships were significant through five competencies (effects ranged from 0.08 to 0.17). The same indirect associations were true for parent–child relationships and teacher-student relationships via five competencies (effects ranged from 0.05 to 0.08), and the proportions of indirect effect in total effect ranged from 31.97% to 53.42%. Thus, except for social awareness in Model 3, four competencies fully mediated the relationships in the models. In Model 3, the indirect effect accounted for 55.00% of the total effect.

**Table 3:** Model fitting indexes and test values.

Model	Mediator	χ^2^	*df*	RMSEA	CFI	TLI
1	Total SEC score	713.10	95	0.05	0.98	0.97
2	Self-awareness	598.56	95	0.04	0.98	0.98
3	Social awareness	294.38	68	0.03	0.99	0.99
4	Self-management	265.04	68	0.03	0.99	0.99
5	Relationship skills	370.13	68	0.04	0.99	0.98
6	Responsible decision-making	214.33	56	0.03	0.99	0.99

**Table 4:** Direct and indirect effects in mediation models.

Model	Mediating path	Indirect effect (β [95% C. I.])	Direct effect (β)	Proportions of indirect effect in total effect
1	PCR → SEC → TSR	0.08 [0.06, 0.10]	0.07^**^	53.06%
	PCR → SEC → PR	0.16 [0.13, 0.18]	−0.02	100.00%
2	PCR → SFA → TSR	0.06 [0.04, 0.08]	0.09^***^	40.82%
	PCR → SFA → PR	0.13 [0.11, 0.16]	0.01	100.00%
3	PCR → SCA → TSR	0.05 [0.03, 0.06]	0.10^***^	31.97%
	PCR → SCA → PR	0.08 [0.06, 0.10]	0.06^**^	55.00%
4	PCR → SM → TSR	0.08 [0.06, 0.10]	0.07^*^	53.42%
	PCR → SM → PR	0.17 [0.14, 0.20]	−0.03	100.00%
5	PCR → RS → TSR	0.07 [0.05, 0.10]	0.07^**^	50.34%
	PCR → RS → PR	0.16 [0.12, 0.20]	−0.02	100.00%
6	PCR → RDM → TSR	0.07 [0.05, 0.10]	0.07^**^	50.34%	PCR → RDM → PR	0.14 [0.11, 0.16]	0.01	100.00%

SFA, Self-Awareness; SCA, Social Awareness; SM, Self-Management; RS, Relationship Skills; RDM, Responsible Decision-Making; PCR, Parent–Child Relationships; TSR, Teacher-Student Relationships; PR, Peer Relationships.

### 4.4 Multi-group analysis

Based on the results of individual difference test, five models were examined with multi-group analysis: Model 1 with gender difference, Model 2 with gender difference, Model 3 with gender and grade differences, Model 5 with gender difference. As shown in Table 5, the configural invariance and the metric invariance of the measurement models were examined (ΔCFI <0.01). In all models, the comparisons of the free and constrained structural paths indicated no significant difference between the male and female cohorts (ΔCFI <0.01).

**Table 5:** Comparisons of measurement and structural models.

Models	χ^2^	*df*	RMSEA	CFI	TLI	Δχ^2^	*Δdf*	*p*	ΔCFI
Model 1 (gender difference)									
1. Configural model	886.75	179	0.05	0.98	0.97	–	–	–	–
2. Metric model	926.56	190	0.05	0.97	0.97	39.81	11	< 0.001	0.001
3. Model with free estimated structural paths	962.22	212	0.05	0.97	0.97	–	–	–	–
4. Model with constrained structural paths	986.45	217	0.05	0.97	0.97	24.23	5	< 0.001	< 0.001
Model 2 (gender difference)									
1. Configural model	691.18	168	0.05	0.98	0.97	–	–	–	–
2. Metric model	710.05	179	0.05	0.98	0.98	18.87	11	0.063	<0.001
3. Model with free estimated structural paths	767.09	201	0.05	0.98	0.97	–	–	–	–
4. Model with constrained structural paths	788.09	206	0.05	0.98	0.973	21.00	5	0.001	<0.001
Model 3 (gender difference)									
1. Configural model	323.56	118	0.04	0.99	0.99	–	–	–	–
2. Metric model	339.41	127	0.04	0.99	0.99	15.85	9	0.070	0.001
3. Model with free estimated structural paths	405.58	145	0.04	0.99	0.98	–	–	–	–
4. Model with constrained structural paths	417.95	150	0.04	0.99	0.98	12.37	5	0.30	0.001
Model 3 (grade difference)									
1. Configural model	597.97	295	0.04	0.99	0.98	–	–	–	–
2. Metric model	673.01	331	0.04	0.98	0.98	75.04	36	< 0.001	0.007
3. Model with free estimated structural paths	939.36	430	0.05	0.98	0.97	–	–	–	–
4. Model with constrained structural paths	957.48	435	0.05	0.97	0.97	18.12	5	0.003	0.002
Model 5 (gender difference)									
1. Configural model	416.95	118	0.04	0.99	0.98	–	–	–	–
2. Metric model	433.34	127	0.04	0.99	0.98	16.39	9	0.060	< 0.001
3. Model with free estimated structural paths	481.11	145	0.04	0.98	0.98	–	–	–	–
4. Model with constrained structural paths	491.55	150	0.04	0.98	0.98	10.44	5	0.064	< 0.001

For the gender differences, parent–child relationships were significantly associated with teacher-student relationships in the female cohort but not in the male cohort in Models 1, 2, 3, and 5 (see Figures 2–4, 6). Therefore, the indirect links between parent–child relationships and teacher-student relationships via the total SEC, self-awareness, social awareness, and relationship skills were full mediating effects in the male cohort, but the partial mediating effects in the female cohort. For Models 1, 2, 3, and 5, the indirect effect accounted for 43.93%, 37.50%, 18.50%, and 40.59% of the total effect, respectively. Besides, in Model 3, parent–child relationships were significantly associated with peer relationships in the female cohort but not in the male cohort. Therefore, social awareness played a full mediating effect in the male cohort, but the partial mediating effect accounted for 47.78% of the total effect in the female cohort.

For the grade differences in Model 3 (see Figure 4), parent–child relationships were significantly associated with teacher-student relationships in the grades 4 and 6 cohorts. Social awareness played a partial mediating effect in grade 4 cohort, accounting for 27.43% of the total effect. In grade 6 cohort, the mediating effect of social awareness was insignificant. For grades 5, 7, and 8 cohorts, social awareness played full mediating effects. Parent–child relationships were significantly associated with peer relationships in grade 5 cohort, and social awareness played a partial mediating effect accounted for 24.47% of the total effect; in other grades, social awareness played full mediating effects.

## 5 Discussion

This study examines the role of five competencies of SEC in interpersonal relationships across early adolescence by exploring data spanning five consecutive grades. Consistent with our hypothesized model, the results of structural equation modeling indicate that students’ parent–child relationships are significantly associated with their teacher-student and peer relationships via their SEC. Furthermore, the multi-group analysis reveals gender and grade differences in the magnitude of these associations. The findings of this study may advance our understanding of students’ SEC proficiency and provide a more nuanced picture of the conditions and interactions in schools and families that foster or hinder students’ SEC proficiency.

### 5.1 The mediating roles of five competencies in interpersonal relationships

Family plays an important role in fostering individual socialization and is closely linked to the future development and career achievements of adolescents (Ermisch and Francesconi, 2001). The current study emphasizes the essential role of parent–child relationships, which are key indicators of the family environment (OECD, 2021). Individuals who perceive their family environment as cohesive, flexible, communicative, and fulfilled, are better at processing emotions (Szcześniak and Tułecka, 2020). The family-of-origin parenting attitudes and practices, combined with the family SES, may shape an individual’s SEC in a certain way (Liu Q. et al., 2020). Individuals who experience warm, loving parent–child connections are more likely to positively view other human interactions and social ties, such as perceiving criticism from others as constructive (Neoh et al., 2021).

Individuals’ SEC represents an essential pillar for students to engage in social interactions, and is associated with three types of interpersonal relationships. SEC of preschoolers could mediate the link between parent–child and teacher-student relationships (Zhang, 2011). Our results confirm the mediating role of five competencies of SEC in early adolescence. Through socialization experiences within the family, adolescents can get along with others in a manner consistent with patterns established in their families (Allen and Loeb, 2015). Chinese parents are also encouraged to consciously or unconsciously guide and nurture children to recognize and regulate emotions, empathize with others, and solve interpersonal problems (Ren and Edwards, 2015).

Students’ four core competencies, i.e., self-awareness, self-management, relationship skills, and responsible decision-making partially mediate the association between parent–child and teacher-student relationships but fully mediate the connection between parent–child and peer relationships. The other core competency, i.e., social awareness, partially mediates the association between the three types of interpersonal relationships. That is, parent–child relationships are indirectly or directly associated with the teacher-student relationship via students’ five competencies. In Chinese traditions, there is a family-like relationship between teachers and students, in which they think of each other as members of an extended family (Yin and Lee, 2012). Chinese teachers are expected to act as parents and interact with students in a parental directing style (Yin, 2015). At the same time, students often consider their teachers as other “parents” in schools, interacting with teachers like parent–child interactions. Parents may influence the way of teacher-student interactions and the children’s perceived teacher-student relationships by reaching out to teachers directly. Meanwhile, students with high SEC are more likely to establish positive relationships with their teachers (Wang et al., 2019), indicating that positive parent–child relationships benefit teacher-student relationships by promoting the development of SEC.

In contrast, the direct associations of parent–child relationships with peer relationships through four competencies are insignificant. Only the direct association through social awareness, which students may be just beginning to sprout in early adolescence, is significant. It is fair to say that SEC plays a strong mediating role in parent–child and peer relationships, similar to the findings of many previous studies (e.g., Kaufman et al., 2020; Lee, 2018; Attili et al., 2010; McDowell and Parke, 2009). Building positive peer relationships is an important part of adolescent development. Unlike relationships with teachers, adolescents tend to seek autonomy in their peer relationships and are reluctant to turn to parents for advice (Smetana and Asquith, 1994) as they develop a sense of self. However, quality parenting behaviors are effective in promoting children’s SEC (Whittaker et al., 2011). Thus, SEC can help adolescents benefit from parent–child relationships in dealing with peer relationships.

### 5.2 Individual differences of five competencies related to demographic factors

The results demonstrate that grade, gender, and family SES are related to adolescents’ SEC. Considering that adolescents’ social arenas are largely confined to home or school, the slight variations in self-awareness, self-management, relationship skills, and responsible decision-making across the five grades may be due to the shortage of socialization experiences. Significant differences in SEC occur with time and experience, since SEC is shaped by experience and learning (Schoon, 2021). Adolescents should be encouraged to assume multiple social roles and engage in different social activities.

It is worth elucidating the grade differences in the mediating role of adolescents’ social awareness in interpersonal relationships. The mediating role was partially present in grade 4 between parent–child and teacher-student relationships, possibly because grade 4 students were relatively naïve and tended to refer more to parent–child relationships when dealing with teacher-student relationships. In grade 6, the mediating effect was insignificant, probably because these participants of the study had just transitioned to middle school after completing 5 years of primary education and were more likely to draw on the parent–child relationship to navigate unfamiliar teachers. Between parent–child and peer relationships, social awareness full mediated in grades 4, 6, 7, and 8 but not in 5. One possible explanation is that the grade 5 participants in the study had recently graduated from primary school and might have been experiencing a lack of social belonging. As a result, they might have temporarily relied more on parent–child relationships for peer relationships. These, further, may suggest that the parent–child relationship is the cradle of the adolescent’s journey toward social relationships and that school experience, as the vital way for adolescent socialization, plays an important role in fostering students’ social awareness.

In terms of family SES, adolescents from economically advantaged families performed higher on SEC measures than their peers, a pattern that is consistent with the literature (e.g., Kuo et al., 2020). This result is not surprising because family SES gaps differentiate individuals’ access to financial and cultural resources that shape experiences, opportunities, social networks, and other aspects (Karus et al., 2012).

### 5.3 Gender differences in five competencies

The results indicate that girls outperform boys in three competencies, i.e., self-awareness, social awareness, and relationship skills during early adolescence. In every cultural setting, individuals learn to conform to certain social expectations are related to gender (Mora, 2012). For example, Hong Kong adolescents manifest the gender gaps in social performance due to the differential social expectations (Shek et al., 2021). Specifically, girls are expected to behave in a nurturing, expressive, and caring way, while boys are expected to be competent by exerting instrumentality, assertion, and competitiveness (Leaper, 2015). In addition, boys’ lag in SEC development is particularly noteworthy. It may be due to the lack of male role models who should model important behaviors for boys (Wood and Brownhill, 2018), as almost 70% of teachers are females in primary and middle schools (MOE, 2021) and that mothers are the primary caregivers for children in China (Liu Q. et al., 2020).

The current results also show gender differences in the association between adolescents’ three competencies (i.e., self-awareness, social awareness, and relationship skills) and three types of interpersonal relationships. Boys’ parent–child relationships tend to be associated with their teacher-student relationships via self-awareness, social awareness, and relationship skills, whereas girls’ parent–child relationships are indirectly and directly associated with teacher-student relationships. Boys’ parent–child relationships tend to be associated with their peer relationships via social awareness, whereas parent–child relationships are indirectly and directly associated with peer relationships for girls. This finding supports the previous view that the relationships between parenting behaviors and adolescents’ SEC development differ across gender (Zhu, 2019).

Adolescents learn about gender role expectations through indirect and direct communication with parents and other family members, which highlights gender differences in the socialization process (Kågesten et al., 2016). This is echoed by the Chinese popular saying “men handle affairs outside the family whereas women manage affairs inside the family” (nan zhu wai, nv zhu nei in Mandarin; Shek and Sun, 2014). In Chinese traditions, men are expected to make friends outside the family, expand their social networks, and participate in social interactions, while women are encouraged to focus their social interactions on parents, siblings, husbands, and children (Chen et al., 2004). In addition, as puberty sets in, girls’ movement may be more restricted due to the adults’ concerns about their developing bodies and emerging sexuality (Mayer et al., 2000). Boys generally have greater freedom to get out of the home and engage in leisure activities (Kågesten et al., 2016). In this case, boys’ SEC may be more developed and employed outside the family, while girls’ SEC may be usually shaped and expressed within the family, resulting in the varying magnitude of parent–child relationships’ effect on other interpersonal relationships in adolescence.

### 5.4 Practical implications

The findings of this study have several important practical implications for parents, teachers and authorities. As parents, first, they should be able to be sensitive and responsive to their children’s emotional needs, not only to take care of their children’s daily life and academic performance, so as to promote students’ SEC proficiency and other social relationships with a good parent–child relationship. A number of studies have shown that Chinese parents are more psychologically controlling of their children than Western parents (e.g., Ng et al., 2014; Qin et al., 2009; Lin and Fu, 1990), which is not conducive to their children’s SEC development (Li and Xie, 2017). Thus, second, Chinese parents need to be more open with their children, treat them more democratically and equally, and encourage them to learn to manage relationships independently, especially girls. Third, Chinese parents need to improve their own parenting behaviors by receiving some professional training, as good parenting behaviors can even diminish the negative impact of disadvantages such as poverty on their children’s SEC development (Ren and Edwards, 2015).

For teachers, first, they should value student development in SEC as much as in their academic achievements. Teachers can contribute to students’ SEC proficiency by improving teacher-student relationships and helping students develop positive peer relationships. Second, the results of the study indicate no significant difference in four competencies performance across grades 4 to 8, so students should be provided with ample opportunities for diverse social activities that allow them to experience different social roles to facilitate their SEC proficiency. Third, teachers should be committed to fostering collaborative efforts among school, family, and the community through consistent goal-oriented actions (Slotkin et al., 2023), including student SEC development programs and parental education programs.

In addition to promoting the role of family-school-community alliances in the development of students’ SEC, authorities should also service children with providing specialized family programs and early childhood intervention programs, especially for infants and toddlers. Studies have shown that the majority of adverse emotional and interpersonal relationships are associated with early family disruption when children are 0–5 years old (Ermisch and Francesconi, 2001). Parents, particularly mothers living in poverty, experience higher levels of stress (Steele et al., 2016), which has been associated with increases in young children’s problem behaviors toward others (Cherry et al., 2019). Early intervention programs can benefit disadvantaged parents and children (Kelly et al., 2024; Love et al., 2002). Research has also shown that access to preschool education is beneficial to the development of children’s SEC (Arapa et al., 2021). The authorities should strengthen the construction of kindergartens so that every young child has the right to early education for PYD.

### 5.5 Study limitations

The limitation of this study, firstly, is the specificity of the theoretical perspective, i.e., the construct and its instruments of five competencies proposed by CASEL and its relevant research were selected among the many theories to collect data. Therefore, the results of the study are also limited to the interpretations within this theoretical perspective. Second, the links between five competencies and interpersonal relationships may be multifaceted and complex. However, the present study examined associations concerning a single direction. Follow-up research could be further extended to explore the possible relationships between these variables in multiple facets and directions. For example, previous studies have found different associations between fathers’ and mothers’ parenting behaviors and their children’s academic, psychological, or social behavioral outcomes (e.g., Cruz et al., 2020; Ward and Lee, 2020). The role of SEC can be further analyzed in the links of father-child and mother–child relationships to other interpersonal relationships, respectively. Third, self-report questionnaires are used to assess students’ five competencies and interpersonal relationships. The results may be influenced by social desirability and confounded by students’ self-evaluation ability. Collecting and integrating data from multiple informants, such as students, parents, and teachers, may increase the data accuracy. Fourth, teacher’s SEC was not included in the model which could be related to students’ proficiency in SEC. A study has shown teacher’s SEC was positively related to adolescents’ prosocial behavior when teachers had strong beliefs about social justice teaching beliefs (Garner and Legette, 2024). Follow-up studies will incorporate this research perspective. Another limitation is that our findings may not generalize to settings outside the specific city where data were gathered. Generalization requires replication in the future study with a more diverse and representative sample. Additionally, the results are discussed and interpreted based on the existing literature without other valuable evidence, such as interviews. Future studies employing qualitative methods would likely offer different and useful perspectives on students’ five competencies and its associations with interpersonal relationships.

## 6 Conclusion

This study adds to existing knowledge about the role of the five competencies of SEC in Chinese students’ interpersonal relationships by analyzing data from five consecutive grades. The results demonstrate that the five competencies in early adolescence vary across family SES, and some of them vary by grade or gender. More importantly, this study provides the structured and comprehensive investigation of the intricate associations among adolescents’ five competencies and three types of interpersonal relationships. Self-awareness, social awareness, and relationship skills fully mediate, and self-management and responsible decision-making partially mediate the association between boys’ parent–child and teacher-student relationships, while five competencies partially mediate the association between girls’ parent–child and teacher-student relationships. Except for social awareness, which partially mediates the association between girls’ parent–child and peer relationships but fully mediates the association between boys’ parent–child and peer relationships, the other four competencies fully mediate the association between girls’ and boys’ parent–child and peer relationships. These findings highlight the importance of parent–child relationships to human SEC proficiency and the pivotal role of SEC in students’ socialization.

## Data Availability

The raw data supporting the conclusions of this article will be made available by the authors, without undue reservation.
