# Supplementary material for: The role of social–emotional competencies in interpersonal relationships: a structural equation modeling approach
Source: Front Psychol. 2024 Oct 3;15:1360467. doi: 10.3389/fpsyg.2024.1360467 (PMC11484989; doi:10.3389/fpsyg.2024.1360467)
Supplement: Supplementary file 1 [file Data_Sheet_1.PDF]

# The Role of Social-Emotional Competencies in Interpersonal Relationships: A Structural Equation Modelling Approach

## *Supplementary Material*

## 1 Supplementary. Computation of SES

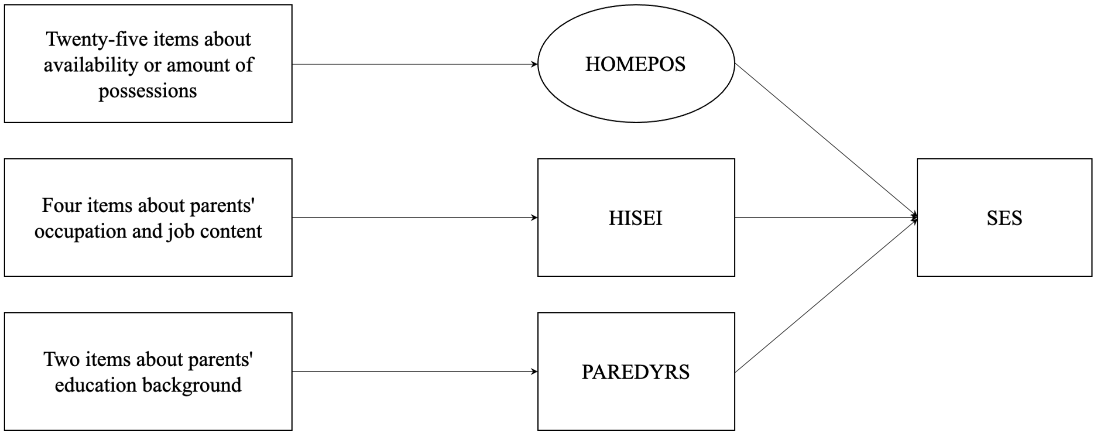


1. HOMEPOS (home possessions) was computed with 25 items by GPCM (generalized partial credit model) and WLE (weighted likelihood estimation) method (RMSEA=0.058, CFI=0.918, TLI=0.909, marginal reliability= 0.865), indicating students' economic capital.

2. Four items were manually coded into parents' ISCO-08 (International Standard Classification of Occupations; International Labour Office, 2012) and then converted into parents' ISEI-08 (International Socio-Economic Index of occupational status; Ganzeboom, 2010). HISEI corresponded to the higher ISEI score of either parent or the only available score, indicating students' social capital.

3. Parents' ISCED (International Standard Classification of Education scheme; UNESCO, 2011) was assessed by two items. HISCED (highest ISCED) corresponded to the higher score of ISCED score of either parent or the only available score. PAREDYRS (Parents' Education Years in Chinese formal education) was converted from HISCED according to the standard (OECD, 2021), indicating students' cultural capital.

4. SES was constructed as the arithmetic mean of the standardized scores of HOMEPOS, HISEI and PAREDYRS. For students with missing data on one out of the three components, the missing variable was imputed by regression on the other two variables. If there were missing data on more than one component, SES was not computed and a missing value was assigned for SES.

## 2 Supplementary. SEC scale description and relevant items

| Scale | | Description | Item |
|---|---|---|---|
| Responsible Decision-Making | .74 | To make caring and constructive choices about personal behavior and social interactions across diverse situations. | # *I blame others when I'm in trouble.*<br>I feel responsible for how I act.<br>I am good at deciding right from wrong. |
| Relationship Skills | .68 | To establish and maintain healthy and supportive relationships and to effectively navigate settings with diverse individuals and groups. | I am good at solving conflicts with others.<br>I get along well with others.<br>I have one or more close friends. |
| Self-Management | .77 | To manage one's emotions, thoughts, and behaviors effectively in different situations and to achieve goals and aspirations. | I am good at waiting for what I want.<br>I can control how I behave.<br>I think before I act. |
| Social Awareness | .70 | To understand the perspectives of and empathize with others, including those from diverse backgrounds, cultures, and contexts. | Others' thoughts are important to me.<br>I think about how others feel.<br>I care about how others feel. |
| Self-Awareness | .88 | To understand one's own emotions, thoughts, and values and how they influence behavior across contexts. | Knowing the emotions I feel.<br>Knowing my strengths and weaknesses.<br>I can handle problems by myself.<br>I can reflect on myself.<br>I am careful of my words and actions. |

#*Note*. Through field test, the overall correlation of this item (a reverse coded) was relatively lower than others and the item was removed in the main study.

## 3 Supplementary. Interpersonal relationships scale description and relevant items

| Scale | | Description | Item |
|---|---|---|---|
| Teacher-Student Relationships | .84 | Feelings about getting along with teachers | Most of my teachers treat me fairly.<br>I get along well with most of my teachers.<br>Most of my teachers are interested in my well-being. |
| Parent-Child Relationships | .82 | Feelings about getting along with parents | I get upset easily with my parents.<br>It is hard for me to talk with my parents.<br>I feel angry with my parents. |
| Peer Relationships | .94 | Feelings about getting along with peers | My friends understand me.<br>My friends accept me as I am.<br>My friends are easy to talk to.<br>My friends respect my feelings. |
